# Beyond adaptive immunity: induction of trained immunity by COVID-19 adenoviral vaccines

**DOI:** 10.1172/JCI166467

**Published:** 2023-01-17

**Authors:** Mihai G. Netea, Leo A.B. Joosten

**Affiliations:** 1 Department of Internal Medicine and Radboud Center for Infectious Diseases, Radboud University Medical Center, Nijmegen, Netherlands.; 2 Department of Immunology and Metabolism, Life & Medical Sciences Institute, University of Bonn, Bonn, Germany.; 3 Department of Medical Genetics, Iuliu Hatieganu University of Medicine and Pharmacy, Cluj-Napoca, Romania.

## Abstract

The COVID-19 pandemic, caused by the SARS-CoV-2 coronavirus, has resulted in much human suffering and societal disruption. The ChAdOx1 nCoV-19 vaccine against COVID-19 has had a crucial role in the fight against the pandemic. While ChAdOx1 nCoV-19 has been shown to induce adaptive B and T cell responses, which protect against COVID-19, in this issue of the *JCI*, Murphy et al. show that this vaccine also induces trained innate immunity. This finding contributes to a better understanding of the complex immunological effects of adenoviral-based vaccines, provides the possibility of clinically relevant heterologous effects of these vaccines, and suggests that other adenoviral-based vaccines may induce trained immunity.

## Live adenovirus as a building block for vaccines

At the end of 2019, a severe pneumonia of unknown origin was identified in Wuhan, China. Soon thereafter, in January 2020, a viral pathogen from the coronavirus family was discovered and termed SARS-CoV-2; the infection caused by it was named coronavirus disease-19 (COVID-19). This highly infectious pathogen led to a major global pandemic, followed in the next three years by hundreds of millions of severe infections and more than 6 million deaths. The healthcare crisis caused by COVID-19, followed by societal and economic disruption, energized global efforts for the development of protective vaccines. Vaccines with high efficacy against infection and severity were rapidly developed, saving millions of lives.

Several technologies have been employed to design and manufacture the COVID-19 vaccines. One very successful vaccine, developed by University of Oxford and AstraZeneca, is based on a chimpanzee adenovirus encoding the SARS-CoV-2 Spike glycoprotein (ChAdOx1 nCoV-19): it has been the most widely used vaccine during the pandemic, with important beneficial effects on the susceptibility to COVID-19 (1). The protective effects of the ChAdOx1 nCoV-19 vaccine against COVID-19 were likely induced by specific neutralizing antibodies and T cell responses (2). The ChAdOx1 nCoV-19 vaccine was not adjuvanted, as it was considered that the adenoviral platform used would have intrinsic bioactivity. Subsequently, however, the use of a live adenovirus as a building block for the vaccine raised an intriguing possibility explored by an elegant study published in this issue of the *JCI* by Murphy and colleagues (3). The authors tested the hypothesis that, in addition to inducing potent specific B and T cell immunity, the ChAdOx1 nCoV-19 vaccine also induced heterologous trained immunity effects (3).

## Inducing trained immunity

Epidemiological studies have shown that certain vaccines, especially those based on live-attenuated microorganisms, are able to induce heterologous protection beyond the target disease (4). The immunological mechanisms mediating these effects likely include induction of cross-reactive T cell responses as well as long-term functional reprogramming of innate immune cells, a process termed trained immunity (5). Increase of antimicrobial function of innate immune cells during induction of trained immunity is antigen independent, which therefore explains the protective effects induced by certain vaccines against other infections than the target disease. The molecular mechanisms underlying trained immunity induced by live-attenuated vaccines are represented by epigenetic and metabolic rewiring of the myeloid cells. Epigenetic changes lead to an increase in chromatin accessibility and an enhanced gene transcription for proteins that are necessary for host defense (5). Among the vaccines shown to induce trained immunity and heterologous protection against infections are both bacterial (e.g., Bacillus Calmette-Guerin [BCG]) and viral (such as measles-containing, oral polio, and influenza) vaccines (6). Based on this prior knowledge, the observation of Murphy and colleagues that the adenovirus-based ChAdOx1 nCoV-19 vaccine induces trained immunity may be somewhat predictable (3).

Murphy et al. provide a systematic analysis of the capacity of the ChAdOx1 nCoV-19 vaccine to induce the various components that characterize trained immunity at different levels (3). The authors showed increased frequency of monocytes in peripheral blood for up to 3 months after vaccination. Moreover, monocyte membrane expression of activation markers and costimulatory molecules, such as HLA-DR, CD40, and CD80, was also enhanced for up to 3 months following vaccination (3). Monocytes had increased expression of glycolysis-associated enzymes, a hallmark of the induction of trained immunity (3, 7). Increased cytokine production capacity after vaccination also characterizes trained immunity. Indeed, upon ex vivo stimulation with unrelated microbial pathogens antigens, monocytes produced increased amounts of IL-1β, IL-6, IL-10, CXCL1, and MIP-1α and decreased quantities of TNF, compared with prevaccine controls. Resting monocytes produced more IFN-γ, IL-18, and MCP-1 for at least three months after vaccination compared with prevaccine controls (3). Only one crucial component of trained immunity responses, the epigenetic reprogramming of gene transcription (8), has not been evaluated by the authors, and this remains to be investigated by future studies.

## Clinical implications and conclusions

**Figure 1:**
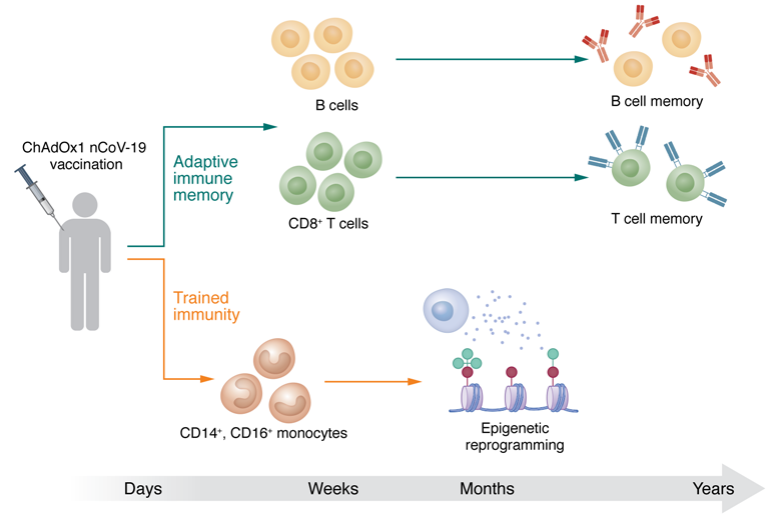
The ChAdOx1 nCoV-19 vaccine induces both classical adaptive immunity and trained immunity against SARS-CoV-2. Within a few weeks of ChAdOx1 nCoV-19 vaccination, T cells and B cells collaborate to generate specific antibodies and activated cells that protect against SARS-CoV-2 infection. Up to three months after vaccination, monocytes show increased frequency in the peripheral blood. They also show increased expression of activation markers, costimulatory molecules, glycolysis-associated enzymes, and cytokines. Increases in these factors are likely a consequence of epigenetic and metabolic reprogramming.

The data presented by Murphy and colleagues are important and relevant at several levels (3). First, the immunological effects of the ChAdOx1 nCoV-19 vaccine likely to extend to other adenoviral-based vaccines. In fact, the data imply that most adenoviral-based vaccines are likely to have a broader immunological effect, comprising both induction of adaptive and innate immune memory (3) (Figure 1). This discovery adds adenoviral-based vaccines to the growing number of vaccines capable of inducing trained immunity processes.

Second, induction of antigen-independent trained immunity could have impactful clinical implications for COVID-19. The correlates of protection for the effect of COVID-19 vaccines have been considered synonymous with neutralizing antibodies against SARS-CoV-2 (9). While induction of specific antibody and T cell responses most likely determine the protection induced by the ChAdOx1 nCoV-19 vaccine against wild-type SARS-CoV-2, they can explain less well the effects of the vaccine against the Omicron strain, which possesses many spike protein differences compared with wild-type SARS-CoV-2. Moreover, the ChAdOx1 nCoV-19 vaccine provides poor protection against Omicron infection, while effectively protecting against severity (10). A similar lack of effect on the total number of infections, but beneficial effects on disease severity, has been reported for other trained immunity–inducing vaccines, such as BCG (11) and MMR (12). Future studies should assess the intriguing possibility that induction of trained immunity may contribute to the beneficial effects of the ChAdOx1 nCoV-19 vaccine and, thus, induce cross-protection against emerging variants.

Third, the induction of trained immunity suggests the possibility that ChAdOx1 nCoV-19 vaccination could induce heterologous protection against other infections beyond COVID-19. Indeed, protection against other pathogens outside the target disease is a characteristic of trained immunity–inducing vaccines (6), and experimental murine studies have shown that intranasally administered adenoviral vectors can induce trained immunity and protect against a different secondary infection (13, 14). Moreover, retrospective analysis of all-cause mortality data of the initial adenoviral-based COVID-19 randomized clinical trial revealed beneficial effects of the ChAdOx1 nCoV-19 vaccine beyond COVID-19 mortality (15), suggesting clinical effects of the vaccine beyond specific protection. However, more studies are needed to be able to conclude whether induction of trained immunity by ChAdOx1 nCoV-19 vaccine has clinical consequences. For example, one aspect that needs attention is the potential for negative hyperinflammatory consequences. Indeed, inappropriate induction of trained immunity is involved in the pathophysiology of many inflammatory diseases (5). As inflammation induces prothrombotic activity, one cannot exclude induction of inappropriate trained immunity activation in some of the rare, yet severe, thrombotic complications of ChAdOx1 nCoV-19 vaccination (16).

Induction of trained immunity by many vaccines has emerged as one of the most active areas of research in the last decade. The intriguing possibility of a role for trained immunity in the vaccination against COVID-19 should be considered along with the mounting evidence for the relevance of this process when considering the immunological effects of vaccines. In addition to the elegant study by Murphy et al. on the ChAdOx1 nCoV-19 vaccine (3), very recent studies have shown that the mRNA vaccines can also induce trained immunity in myeloid cells (17, 18). This finding implies that both platforms on which COVID-19 vaccines are based have heterologous immunological effects, which should be taken into account when assessing their clinical impact.

Murphy et al. (3) shed light on the complexity of the immunological effects of ChAdOx1 nCoV-19. The demonstration of induced trained immunity by ChAdOx1 nCoV-19 opens the door for a better understanding of COVID-19 vaccines. Nevertheless, important questions remain and should be investigated in future studies. (a) What are the epigenetic mechanisms through which adenoviral (and mRNA-based) COVID-19 vaccines induce trained immunity? (b) Is trained immunity also induced in cell types other than monocytes? (c) What are the clinical consequences of the induced trained immunity? Only when these questions have been answered will clinicians be able to glean the full potential of these vaccines in practice.

## Version 1 01/17/2023

Electronic publication
